# Editorial: Cognitive Dysfunctions in Psychiatric Disorders: Brain-Immune Interaction Mechanisms and Integrative Therapeutic Approaches

**DOI:** 10.3389/fnint.2021.649425

**Published:** 2021-02-18

**Authors:** Haiyun Xu, Weiwen Wang, Bart Ellenbroek, Zili You

**Affiliations:** ^1^School of Mental Health, Wenzhou Medical University, Wenzhou, China; ^2^CAS Key Laboratory of Mental Health, Institute of Psychology, Beijing, China; ^3^Department of Psychology, University of Chinese Academy of Sciences, Beijing, China; ^4^School of Psychology, Victoria University of Wellington, Wellington, New Zealand; ^5^School of Life Science and Technology, Center for Informational Biology, University of Electronic Science and Technology of China, Chengdu, China

**Keywords:** brain, cognitive dysfunction, immune regulation, mental disorder, neuroinflammation

This Research Topic, consisting of 13 research and review articles, aimed to deepen our understanding of the brain-immune interaction mechanisms underlying cognitive dysfunction in psychiatric disorders and help to develop better integrative therapeutic approaches of the five human research articles, the study by Yang Z. et al. reported that patients with schizophrenia performed poorly compared to healthy controls in all the cognitive tasks in the repeatable battery for the assessment of neuropsychological status, which consists of 12 subtests measuring the cognitive domains of immediate memory, visuospatial/constructional, language, attention, and delayed memory (Randolph et al., 1998). In addition, patients with schizophrenia showed lower plasma GDF-11 (growth differentiation factor 11) relative to that in healthy controls. Moreover, there were significant negative correlations between GDF-11 level and the PANSS total score, positive symptoms score, negative symptoms score, or general psychopathological score in the patients, whereas plasma GDF-11 level was positively associated with the immediate memory and delayed memory. The results of this study added evidence associating GDF-11 to the cognitive impairments (CI) in schizophrenia patients thus linking this TGF-ß superfamily member to the pathophysiology of schizophrenia.

Numerous neuropsychological studies demonstrate a broad range of measurable cognitive deficits in patients with schizophrenia, including impairments in attention, working memory (WM), episodic memory, processing speed, and executive functions (Xu et al., 2011; Kahn and Keefe, 2013). Here Xin et al. investigated how acute stress influences human WM functions using a well-validated neural measure of visual–spatial WM storage. They found: (1) the participants performed the visual WM task better after the control treatment than after the CPS (cold pressor stress) treatment, (2) the subjects showed a late CDA (contralateral delay activity) amplitude in the two-red-items condition after the CPS treatment, which was significantly larger than that after the control treatment, and (3) N2pc components showed significant disparity across three types of stimuli arrays after control treatment, which disappeared in the CPS session. These results demonstrate that acute stress impaired individuals' cognition processes of object individuation and maintenance in visual–spatial WM, as well as in task performance.

There is increasing evidence supporting the association of depression with overall CI among people living with HIV (PLWH), and in the cognitive domains of processing speed, executive function, as well as learning and memory (Rubin and Maki, 2019). Moreover, CI has been linked to incomplete HIV suppression in the CNS despite effective antiretroviral therapy (Zayyad and Spudich, 2015; Saylor et al., 2016). Here Rubin et al. examined the contribution of early immune signatures to cognitive performance among HIV-infected, virally suppressed women (HIV+VS) and in HIV-uninfected (HIV–) women. They showed that leukocyte influx into brain was a major contributor to CI in HIV+VS women, while T cell exhaustion, microglial activity, and cytokine signaling predicted both global and domain-specific performance for HIV– women.

Non-invasive neuromodulatory techniques such as rTMS (repeated transcranial magnetic stimulation) and tDCS (transcranial direct current stimulation) have been demonstrated to help prevent and ameliorate stress, with effects on emotion, cognition, behavior, as well as autonomic nervous system (Schestatsky et al., 2013). Here Castelo-Branco and Fregni provided a comment on the potential tDCS application on preventing and treating stress-related symptoms during social isolation, while addressing the feasibility and efficacy of home-based tDCS. Furthermore, Carvalho et al. designed a study protocol for a parallel, randomized, triple-blind, sham-controlled clinical trial in which a total of 90 drug-naïve, first-episode MDD patients (45 per arm) will receive a 6-weeks of CBT combined with either active or sham tDCS applied to DLPFC. The primary outcome is depressive symptoms improvement while the secondary aim is to test whether CBT combined with tDCS can engage the proposed mechanistic target of restoring the prefrontal imbalance and connectivity through the bilateral neuro-modulation of the DLPFC.

High validity disease-related animal model is a powerful tool to explore the immune regulation of cognitive dysfunction and underlying brain mechanism. Using a classic animal model of schizophrenia induced by social isolation, Zhou et al. reported prepulse inhibition (PPI) deficits in C57BL/6J mice after social isolation for 5 weeks during PND21-56. And the CX3CR1 protein level was increased in the medial prefrontal cortex, nucleus accumbens and hippocampus of the isolated C57BL/6J mice. But the same protocol had no effect on PPI in the CX3CR1^−/−^ mice, which were stress resistant as shown in a previous study (Winkler et al., 2017). CX3CR1 and its ligand CX3CL1 directly modulate the function of microglia (Wolf et al., 2013). Therefore, the authors suggested that the social isolation-induced PPI deficit in mice is CX3CR1-dependent.

Relevant to the above animal study, Zhang et al. used a mouse model of MIA (maternal immune activation) by the viral dsRNA analog poly(I:C) to mimic the effects of inflammation during pregnancy. They found that mRNA expression of the genes SRY-related HMG-box 10, myelin-associated glycoprotein, and transferrin were generally reduced in the limbic system of the hemispheres of prenatal poly(I:C)-exposed mouse offspring. The results provided evidence that prenatal exposure to poly(I:C) elicits profound and long-term changes in transcript level and spatial distribution of myelin-related genes in multiple neocortical and limbic regions, which play prominent roles in learning, memory, and social interaction.

The evidence for the involvement of microglia and oligodendrocytes (OLs) in the pathogenesis of psychiatric disorders also came from another animal model, the cuprizone-exposed mouse. Cuprizone (CPZ) is a chemical chelator toxic to mitochondria (Pasquini et al., 2007; Acs et al., 2013). In comparison to the other animal models of schizophrenia, the brain neuropathology of CPZ-exposed mice involves mitochondrial dysfunction, neuroinflammation, and white matter damage, three essential components in the pathogenesis of schizophrenia (Najjar and Pearlman, 2015; Xu et al., 2016). Here Yang L. et al. reported that deep rTMS for 4 weeks during the CPZ-exposure period effectively blocked the behavioral changes, myelin breakdown, and OL loss in CPZ-fed mice while inhibited microglia activation in the lesion sites and regulated the expression of inflammatory cytokines of IL-1ß, IL-6, and IL-10 in the same brain regions. These data added experimental evidence for the beneficial effects of deep rTMS in treating behavioral abnormalities such as working memory and improving the associated neuropathologic damage like microglia activation and OL loss in the brain.

The senescence accelerated mouse prone strain-8 (SAMP8) is an established and widely used model that features accelerated aging (Takeda, 1999) and shows spatial learning impairments from 12 weeks of age and spatial memory deficits commencing from 16 weeks of age (Flood and Morley, 1998; Cheng et al., 2008). Here, Lam et al. compared from a young age of 6 weeks cognitive and motor performance of SAMP8 in an olfactory-visual spatial water maze task administered longitudinally. In classic MWM (Morris-Water-Maze), it took much more time for SAMP8 to reach a rescue platform compared to age-matched SAMR1 (senescence accelerated mouse resistant-strain 1) mice. And this strain difference was apparent from as young as 6 weeks of age. In the olfactory-visual water maze, this strain difference in latency time to reach the rescue platform was markedly greater compared to the classic MWM, indicating a significant olfactory processing deficit in the SAMP8 strain. The findings complement the extant literature in SAMP8 mice that demonstrate CNS physiological aberrations preceding loss of spatial and associative memory.

The last, but not least, an animal study by Wang et al. reported that Notoginsenoside R1 (NR1, the main component with cardiovascular activity in Panax notoginseng) effectively blocked the spatial and cognitive impairment in C57BL/6 mice exposed to acrylamide, a common chemical having chronic neurotoxic effects. Moreover, NR1 protected against the acrylamide-induced neurotoxicity by upregulating thioredoxin-1 in PC12 cells and mice. In the meanwhile, the autophagy-related proteins-like Cathepsin D, LC3 II, lysosomal-associated membrane protein 2a, and integrin alpha V were restored to normal levels by NR1 in both PC12 cells and mice. The results suggest a potential application of NR1 in treating acrylamide-induced neurotoxicity.

As mentioned above, inflammatory responses and immunity are important players in the development and onset of depression. Therefore, modulation of inflammation may mitigate depressive behavior in depressive patients. Here Kim et al. reviewed the therapeutic effects of GLP-1 (glucagon-like peptide-1) on depression from a variety of perspectives. They summarized evidence showing that GLP-1 attenuates the process of neuroinflammation and protects neurons and glia under oxidative stress conditions in the depressive brain, described the role of GLP-1 in neurotransmitter homeostasis in the depressive brain in which GLP-1 improves neurotransmitter balance, reviewed the role GLP-1 in promoting neuronal differentiation and neural stem cell proliferation in the depressive brain, and summarized studies showing the improvement of impaired cognitive function in subjects by GLP-1.

The over-activation of microglia has been posited to contribute to suicidal behavior (Calcia et al., 2016; Mondelli et al., 2017). Therefore, chronic microglial activation may influence the interaction between the stress and diathesis components of the stress diathesis model of suicidal behavior by contributing to an increased vulnerability to suicidal tendencies. Here Baharikhoob and Kolla reviewed the findings from human post-mortem and neuroimaging studies reporting a relationship between microglial activation and suicidal behavior and updated the clinical model of suicidal behavior to integrate the role of microglia.

Another category of psychiatric disorders is dementia. Of the conditions leading to dementia, the most common one is Alzheimer's disease (AD, 50–75%) followed by vascular dementia (20%), dementia with Lewy bodies (5%), and frontotemporal lobar dementia (5%) (Prince et al., 2013). In addition, patients with amyotrophic lateral sclerosis (ALS) may also experience cognitive impairment (Beeldman et al., 2016). Therefore, this Research Topic included a review article by Guan and Han summarizing recent research developments regarding the NLRP3 inflammasome and its role in AD, PD, and ALS. In AD, Aß induces NLRP3 activation followed by ever-increasing production of IL-1ß and the other proinflammatory cytokines from microglia. In this manner, the neurotoxic effects of Aß on brain neurons are amplified (Heneka et al., 2013). In patients with PD, fibrillar a-syn induces NLRP3-caspase-1 complex activation which in turn causes the truncation and aggregation of a-syn. Then a-syn enters microglial cells in an endocytosis-dependent manner and subsequently activates two innate receptors in the TLR2 (TLR4)/NF-kB and NLRP3/caspase-1 pathways (Gustot et al., 2015; Fan et al., 2016). In ALS, microglia and astrocytes switch from a neuroprotective to a proinflammatory phenotype by releasing potentially neurotoxic cytokines. Importantly, inhibition of NLRP3 in animal models reduces the inflammatory response, decreases abnormal protein deposition, and corrects behavioral abnormalities associated with neurodegenerative diseases, suggesting that targeting NLRP3 inflammasome is a plausible therapeutic approach to treating such psychiatric disorders.

In sum, the collection highlights the wide range of technological strategies and research models available, each with its own advantages. These studies not only advance our understanding of the brain-immune interaction mechanisms underlying cognitive dysfunction in psychiatric disorders, but also help develop promising avenues to improve cognitive impairments in patients with psychiatric disorders.

## Author Contributions

HX wrote the draft. WW, BE, and ZY read and revised the draft. All authors approved the publication of this editorial.

## Conflict of Interest

The authors declare that the research was conducted in the absence of any commercial or financial relationships that could be construed as a potential conflict of interest.
